# Abnormalities of cell packing density and dendritic complexity in the MeCP2 A140V mouse model of Rett syndrome/X-linked mental retardation

**DOI:** 10.1186/1471-2202-11-19

**Published:** 2010-02-17

**Authors:** Garilyn M Jentarra, Shannon L Olfers, Stephen G Rice, Nishit Srivastava, Gregg E Homanics, Mary Blue, SakkuBai Naidu, Vinodh Narayanan

**Affiliations:** 1 Neurology Research Department, Barrow Neurological Institute, 350 W Thomas Rd, NRC 438, Phoenix, AZ, 85013, USA; 2 Departments of Anesthesiology and Pharmacology & Chemical Biology, University of Pittsburgh, 6060 Biomedical Science Tower 3, Pittsburgh, PA, 15261, USA; 3 Neuroscience Laboratory, Kennedy Krieger Institute, 707 North Broadway, Baltimore, MD, 21205, USA; 4 Departments of Neurology and Pediatrics, Kennedy Krieger Institute, 707 North Broadway, Baltimore, MD, 21205, USA; 5 School of Life Sciences, Arizona State University, University Drive and Mill Ave, Tempe, AZ, 85287, USA

## Abstract

**Background:**

Rett syndrome (RTT), a common cause of mental retardation in girls, is associated with mutations in the *MECP2 *gene. Most human cases of *MECP2 *mutation in girls result in classical or variant forms of RTT. When these same mutations occur in males, they often present as severe neonatal encephalopathy. However, some *MECP2 *mutations can also lead to diseases characterized as mental retardation syndromes, particularly in boys. One of these mutations, A140V, is a common, recurring missense mutation accounting for about 0.6% of all MeCP2 mutations and ranking 21^st ^by frequency. It has been described in familial X-linked mental retardation (XLMR), PPM- X syndrome (Parkinsonism, Pyramidal signs, Macroorchidism, X-linked mental retardation) and in other neuropsychiatric syndromes. Interestingly, this mutation has been reported to preserve the methyl-CpG binding function of the MeCP2 protein while compromising its ability to bind to the mental retardation associated protein ATRX.

**Results:**

We report the construction and initial characterization of a mouse model expressing the A140V MeCP2 mutation. These initial descriptive studies in male hemizygous mice have revealed brain abnormalities seen in both RTT and mental retardation. The abnormalities found include increases in cell packing density in the brain and a significant reduction in the complexity of neuronal dendritic branching. In contrast to some MeCP2 mutation mouse models, the A140V mouse has an apparently normal lifespan and normal weight gain patterns with no obvious seizures, tremors, breathing difficulties or kyphosis.

**Conclusion:**

We have identified various neurological abnormalities in this mouse model of Rett syndrome/X-linked mental retardation which may help to elucidate the manner in which *MECP2 *mutations cause neuronal changes resulting in mental retardation without the confounding effects of seizures, chronic hypoventilation, or other Rett syndrome associated symptoms.

## Background

Rett syndrome (RTT; OMIM #312750) is an X-linked dominant neurological disorder affecting predominantly females [1,2] and has an estimated prevalence of 1 in 10,000-15,000 females [3]. In patients with RTT, symptoms are generally recognized around 6 to 18 months of age following an apparently normal period of early development. Regression of previously acquired skills and deceleration of head growth are key features of the disease in conjunction with stereotypic hand movements. Ultimately, affected individuals frequently display cognitive, social, and language impairments as well as a variety of serious motor and breathing abnormalities, seizures, microcephaly, gastrointestinal dysfunction, scoliosis, kyphosis, and rigidity [2-4]. Pathological studies of human RTT cases have shown a reduction in brain size, and an increase in cell packing density [5,6]. These studies have also noted a significant reduction in the dendritic branching of neurons in specific layers of the frontal, motor, temporal and limbic cortex [6-9].

Most cases of RTT are caused by point mutations, insertions, duplications, or deletions in the *MECP2 *(methyl-CpG binding protein 2) gene [1,4,10]. Over 300 distinct *MECP2 *mutations have been described and certain mutations occur with high frequency (T158M, R168X, R255X, R270X, R294X, R306C, R133C, and R106W) (IRSA MeCP2 Variation Database, http://mecp2.chw.edu.au/mecp2/). At least two important functional domains within the MeCP2 protein have been identified. The first is a methyl-CpG binding domain (MBD) which binds specifically to methylated CpG- dinucleotides and the second is a transcriptional repressor domain (TRD), which interacts with a transcriptional repressor complex [11,12]. While widely described in the past as a transcriptional repressor, a recent report indicates that MeCP2 may also be a transcriptional activator, and may actually function more often as an activator than a repressor of transcription [13]. It is also believed to participate in recruiting chromatin remodeling and co-repressor proteins [11,12], and [14]. MeCP2 acts to modulate the transcription of many genes, one of which, BDNF [13,15,16], is particularly relevant to diseases causing mental retardation as it is heavily involved in synaptic plasticity [17] and influences disease progression in *Mecp2 *mutant mice [18].

In addition to the classic or variant RTT phenotypes in females, *MECP2 *gene mutation has also been described in males [19-21], sometimes causing a severe and fatal early onset encephalopathy [22]. One specific mutation of MeCP2, A140V, does not result in a severe phenotype or in early lethality. While not resulting in classical RTT features, this mutation has been reported to cause X-linked mental retardation (XLMR) and neuropsychiatric syndromes as well as PPM-X syndrome (OMIM #300055; Parkinsonism, Pyramidal signs, Macroorchidism, X-linked mental retardation) [23-26]. The A140V mutation is a recurring mutation within the MBD of MeCP2 accounting for about 0.6% of all reported MeCP2 mutations (IRSA MeCP2 Variation Database, http://mecp2.chw.edu.au/mecp2/). This mutation was first reported in a female with mild mental retardation, her daughter with mild mental retardation, and her four sons with severe mental retardation [23]. In a cohort of mentally retarded patients negative for expansion of the CGG repeat at the *FMR1 *locus, Couvert et al found two sporadic cases of A140V mutation [21]. A three-generation family with five affected males (MRX 79) was shown to carry the A140V mutation [26]. The A140V mutation has also been reported in a young boy with language disorder and schizophrenia [27]. In addition to the previously listed symptoms, dysarthric speech, mild dysmorphic features, spasticity, gait abnormalities, mild microcephaly, trophic changes of the legs, distal atrophy in the limbs, kyphoscoliosis, ankle clonus, and abnormal deep tendon reflexes are also symptoms associated with this mutation [23]. Thus, the A140V MeCP2 mutation is an important cause of XLMR and developmental disorders and is somewhat distinct clinically from classic RTT.

It has been predicted based on structural data that the A140V mutation, located in the α1 helix of the MBD, could potentially affect the structure of the MeCP2 MBD and therefore the methyl-CpG DNA binding function. Ohki, et al. [28] speculate that due to its bulky side chain, the substituted valine may cause reduced DNA binding affinity and Orrico, et al., [25] using secondary structure prediction programs, proposed that the A140V mutation shortens the α1 helix by half, potentially resulting in an alteration of function. Although many *MECP2 *gene mutations do affect the methyl-CpG DNA binding function of the MeCP2 protein, the A140V mutation has been reported not to, despite predictions based on its location within that functional domain [29]. Indeed, MeCP2 A140V protein localizes to heterochromatic foci, containing abundant methyl- CpG sites, as efficiently as wild type (WT) MeCP2. In addition, DNA binding assays in cultured cells have demonstrated that the A140V mutation does not affect binding to methylated DNA [29,30]. Nevertheless, another interesting property of MeCP2 A140V protein was discovered in studies of transcriptional activity in transfected cells. MeCP2 A140V repressed transcriptional activity from methylated promoters at levels comparable to WT and from unmethylated promoters at a level approximately 40% higher than WT MeCP2 [30]. This raises the possibility that higher repressive activity by MeCP2 A140V may affect the normal expression of unmethylated genes.

The A140V mutation is also reported to directly affect the ability of MeCP2 to bind to the ATRX protein [29], a helicase/ATPase believed to be involved in chromatin remodeling [22]. Alpha-thalassemia X-linked mental retardation syndrome (ATRX; OMIM #301040) is caused by mutation of the *ATRX *gene. In addition to the hematologic abnormality alpha-thalassemia, *ATRX *gene mutations also result in mental retardation, microcephaly, dysmorphic features, genital and renal abnormalities, growth deficiency, seizures, spasticity, gastrointestinal dysfunction, and kyphoscoliosis and other skeletal abnormalities [31-34]. Many of the symptoms of ATRX overlap those seen in patients with a classic RTT mutation of the *MECP2 *gene. There are also clear differences, the presence of dysmorphic features in *ATRX *mutations for example. Interestingly, the A140V mutation reproduces symptoms of both conditions while still being somewhat distinct from each of them. This may provide a glimpse into the mechanism underlying each of these conditions. Currently there are no complete ATRX knock out mouse models due likely to the described embryonic lethality in males [35]. Tissue specific conditional inactivation of ATRX results in enhanced cell death during corticogenesis with early lethality (forebrain specific inactivation) [36] and interneuron defects in the retina (retina specific inactivation) [37].

A variety of mouse models of RTT have been previously developed and tested. One of the first *Mecp2 *mutant mouse models generated lacked exons 3 and 4. This model demonstrated that MeCP2-null animals were viable and did not have a specific initial phenotype. Between the ages of 3 and 8 weeks these animals (null males) developed abnormalities of gait and breathing, ultimately leading to early death at around 2 months of age [38]. A second MeCP2-null mouse was generated by deletion of Exon 3. Male mice appeared healthy for the first few weeks of age but developed abnormal movements, motor control, and breathing by 5 weeks of age with death occurring around 10 weeks. Female mice in this study exhibited similar symptoms but with a time course delayed by months [39].

Shahbazian et al. [40] generated mice with truncation of the C-terminal third of the coding region (308/y mouse) by introducing a stop codon following amino acid 308, which is located at the C terminal end of the transcriptional repressor domain. Male mutant mice survived to at least 1 year of age and developed a variety of symptoms including tremor, myoclonic seizures, kyphosis and stereotyped forelimb movements. The motor performance of these mice declined with age. Histological studies of the brain were normal and fear conditioning and spatial learning were normal, however, the mice did display enhanced anxiety and an abnormal corticosterone stress response [41]. Recently, a knock-in model of RTT (R168X) was generated, reproducing a common non-sense mutation of MeCP2 found in human cases. As with other RTT model mice, male R168X mice die early at around 12 weeks of age, but display symptoms suggestive of RTT such as breathing irregularities, hypoactivity, and hind limb atrophy and clasping. Female heterozygous mice show similar symptoms but not until around 6 months of age [42]. As reported in pathological studies of human RTT brain tissues [5,6], abnormalities of brain size [39,43] and cell packing density have been observed in mouse models of RTT [39,44,45]. Abnormalities of dendrite branching have also been reported in some Rett syndrome mouse models [44,45]. Much of the testing in mouse models has been done using hemizygous males to avoid the phenotype variability resulting from random X inactivation in females, which is considered to influence phenotype in human RTT patients. We have performed our initial tests in males for this reason and because A140V is more commonly recognized in longer surviving male patients.

The pathogenesis of the neurological phenotype in RTT remains unclear, and continued study of mouse models will improve our understanding. The various mouse models recapitulate the symptoms of RTT in many ways but are often distinct from each other. Although much has been learned about RTT through the study of existing mouse models, the A140V model we have created is unique. It reproduces a recurring missense mutation seen in humans and, in males, results in an XLMR phenotype (rather than classical RTT). In addition, due to the long life span of mutant male mice, developmental studies can be performed. We expect that by a careful analysis of the A140V mouse, we will better understand the important roles that MeCP2 plays in the pathogenesis of developmental brain disorders that result in mental retardation. Here we describe the construction and initial characterization of brain abnormalities in the MeCP2 A140V mutant mouse model of RTT/XLMR. In addition to being a model for these two diseases, the A140V mutant mouse permits the study of a novel mechanism by which MeCP2 mutation might affect neural development; its interaction with the ATRX chromatin remodeling protein.

## Results

### Generation of the MeCP2 A140V mutant mouse

**Figure 1:**
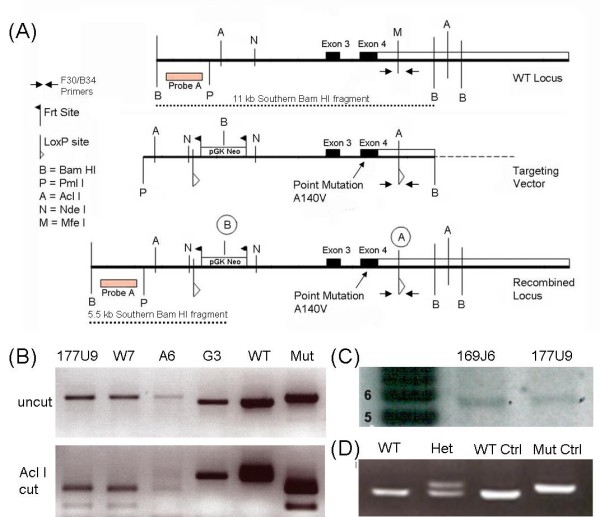
**Generation of the MeCP2 A140V "knock-in" mouse**. (1A) Diagram showing the WT *Mecp2 *locus, the A140V targeting vector, and the recombined MeCP2 A140V locus used to create the knock-in mouse. (1B) PCR demonstrating ES cell DNA positive (177U9, W7, and A6) and negative (G3) for homologous recombination at the 3' end of the targeting vector. Lanes "WT" and "Mut" are plasmid DNA controls demonstrating the WT and A140V mutant PCR fragments (Upper Panel). The PCR fragments were digested with Acl I which cuts only the mutant PCR fragments, confirming the presence of the A140V mutation and producing a double band (1B Lower Panel). (1C) Southern blot analysis Bam HI digested ES cell DNA. A 5.5 kilobase pair (kbp) band was detected confirming homologous recombination at the 5' end of the targeting vector. The ladder on the left is in kbp. (1D) PCR genotyping results of F1 generation females. DNA from a WT female produces a single band while DNA from a heterozygous female (Het) produces two bands. The WT and Mut lanes show control plasmid reactions.

The A140V mouse model of RTT/XLMR was generated by introducing a point mutation (a C to T change at nucleotide position 601 of the MeCP2-e2 mRNA, NCBI Reference Sequence: NM_010788.3) that results in the A140V missense mutation of MeCP2 protein. The WT locus, targeting vector, and recombined locus, are diagrammed in Figure 1A; also shown in Figure 1A is the location of Probe A (external to the targeting vector) used for Southern blot analysis as well as the Southern blot Bam HI fragments. In addition, the location of the genotyping primers (F30/B34) is indicated. Results of a PCR based test of ES cell DNA to detect homologous recombination at the 3'-end of the targeting vector are shown in Figure 1B (Upper Panel). The WT allele yields a 322 bp fragment while the mutant allele yields a 362 bp fragment. As a confirmatory test these fragments were digested with Acl I which cuts the mutant allele but not the WT allele, yielding a double band of 227 bp and 135 bp (Figure 1B Lower Panel). Southern blot analysis of Bam HI digested ES cell DNAs detects a 5.5 kbp band, confirming homologous recombination at the 5'-end of the targeting vector (Figure 1C). ES cell clones that were positive by PCR (3'-end) and Southern blot (5'-end) were then sequenced to verify that they were hemizygous for the A140V mutation (data not shown) and two of these clones were used for blastocyst injection. Shown in Figure 1D are PCR genotyping results of F1 females: heterozygous females produce two bands and WT females produce a single (smaller) band. Plasmid controls show either the single smaller band (WT) or single larger band (mutant). A chimeric male created from the 177U9 ES cell clone was used to produce heterozygous female founders for the MeCP2 A140V mouse colony.

### Mutant Mecp2 mRNA is expressed in brain tissue

**Figure 2:**
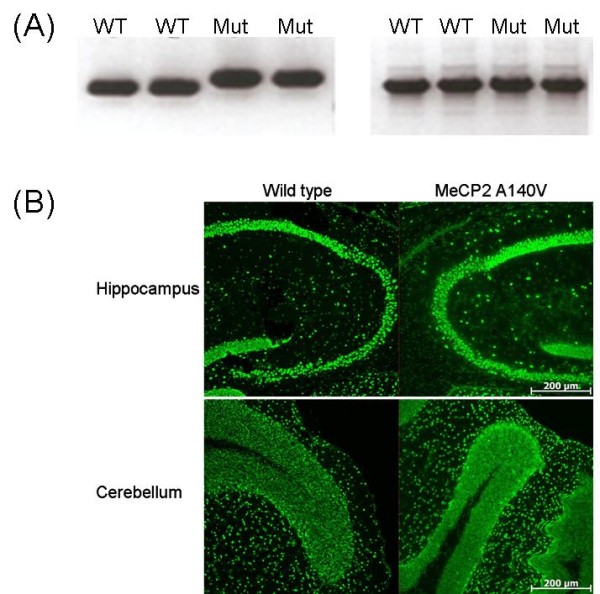
***Mecp2 *mRNA and protein are present in the brains of A140V mutant male mice**. (2A) Total RNA was extracted from the cerebella of mice and used for cDNA synthesis. RT-PCR confirmed the presence of *Mecp2 *mRNA in the cerebella of 2 WT and 2 A140V (Mut) mice (Left Panel). The mutant mRNA species results in a larger fragment by PCR than the WT mRNA species. A second RT-PCR reaction amplifying a different segment of the cDNA containing the A140V mutation site confirmed that there was no contamination of the total RNA used for the cDNA synthesis with genomic DNA. WT and A140V (Mut) cDNA showed only the presence of a band of the correct size for cDNA PCR product (Right Panel). (2B) Immunohistochemistry on frozen brain slices demonstrates the presence of MeCP2 protein in the brain of an A140V hemizygous mouse. Staining in the hippocampus and the cerebellum are similar in the WT and A140V mice using an antibody recognizing the C-terminus of MeCP2. Scale bars are 200 μm.

We confirmed that only mutant *Mecp2 *mRNA was present in brain tissue of male hemizygous animals by reverse transcription PCR (RT-PCR). cDNA was prepared from cerebellar total RNA extracted from male animals (2 A140V mutant and 2 WT controls), and tested by PCR (with primers F30 and B34, located within the 3'-UTR). As shown in Figure 2A (Left Panel) mutant animals display only the larger fragment while WT animals display only the smaller fragment. This result also confirms the presence of the *Mecp2 *3'-UTR. A different segment of cerebellar *Mecp2 *cDNA (one that includes the A140V site) was then amplified to confirm that there was no genomic DNA contamination of the cDNA used in these tests. Samples from both WT and mutant animals showed only the cDNA band (shown) and not a larger genomic DNA band (data not shown), thus the cDNA samples were free of genomic DNA contamination (Figure 2A Right Panel). Sequencing performed on purified cDNA from these bands confirmed the A140V mutation (data not shown).

### MeCP2 protein is expressed in the brain of A140V mutant animals

As this is not a knock-out or truncating model, it was expected that the full length MeCP2 protein would be present in the brains of hemizygous male mice. Immunostaining in brain slices using an anti-MeCP2 antibody directed against the C-terminus of the protein demonstrated the presence of the MeCP2 protein in the brain tissue of hemizygous mice as well as wild type control mice (Figure 2B). A peptide blocking assay confirmed the specificity of the antibody (data not shown). The mutation does not appear to have affected the nuclear or brain regional distribution of MeCP2 protein.

### Mecp2 mRNA levels in wild-type and A140V mouse cerebellum by qRT-PCR

**Figure 3:**
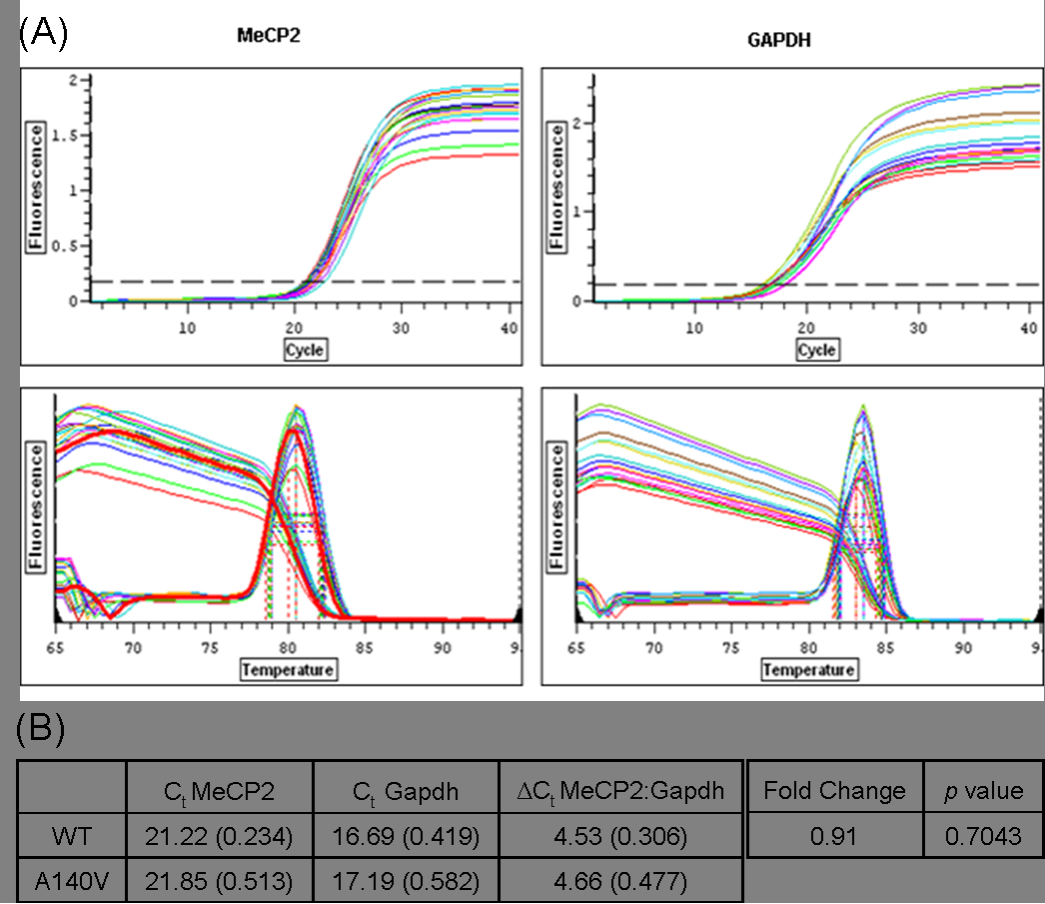
***Mecp2 *mRNA levels are similar in WT and A140V mouse cerebellum by qRT-PCR**. (3A) Quantitative real time PCR (qRT-PCR) reactions were performed using PCR assays for *Mecp2 *and *Gapdh*. qRT-PCR data graphs (Upper Panels) and melting curves (Lower Panels) for *Mecp2 *and *Gapdh *are shown. (3B) A table of C_t _(cycle threshold) values is shown including mean and standard deviation (in parentheses) and ΔC_t _used in the calculation of fold change (F) in mRNA levels. F (*Mecp2*:*Gapdh*) = 0.91.

Total RNA was extracted from the cerebellum of three A140V male mice and three WT male littermates (6 months of age). Quantitative real time PCR (qRT-PCR) reactions were performed using PCR assays for *Mecp2 *and *Gapdh *from SA Biosciences (Frederick, MD). The primer set for *Mecp2 *detects both the e2 and e1 isoforms of *Mecp2*. Figure 3 shows data graphs for *Mecp2 *and *Gapdh *(qRT-PCR and melting curves) (3A) as well as a table of C_t _(cycle threshold) values including mean and standard deviation (in parentheses), and ΔC_t _used in the calculation of fold change (F) in mRNA levels, comparing A140V to WT (3B). Fold change between mutant and wild-type is calculated using the ΔΔC_t _method (F = 2 exp [- (ΔC_t_.mutant - ΔC_t_.wild-type)]). In the cerebellum, fold change in *Mecp2 *mRNA levels (A140V: WT; normalized to *Gapdh *levels) was 0.91 (*p *= 0.7043). This demonstrated that in the cerebellum of WT and mutant 6 month old animals we were unable to detect a significant difference in the abundance of WT and mutant steady state *Mecp2 *mRNA (respectively). This suggests that the A140V mutation does not significantly affect transcription or mRNA stability.

### There are no obvious phenotypic abnormalities of the A140V mice

**Figure 4:**
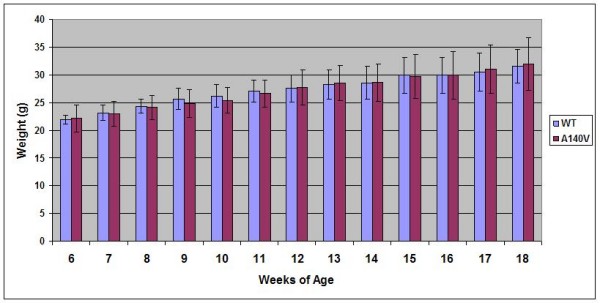
**MeCP2 A140V male mice show similar somatic growth patterns to WT controls**. Male WT and MeCP2 A140V mice were weighed weekly between the ages of 6 and 18 weeks. No significant difference was found between the mean weights of the two groups. Error bars indicate one standard deviation.

No obvious phenotypic differences between A140V and WT male mice were noted. Somatic growth between 6 and 18 weeks of age in mutant animals was similar to WT controls (Figure 4). We have not observed abnormalities in body morphology (such as kyphosis) in animals observed up to 14 months of age. Necropsy of mutant animals has not revealed any gross organ pathology. No seizures or tremors have been observed in these animals. Female heterozygous mice displayed a normal level of fertility although attempted breedings of male hemizygous mice have thus far been unsuccessful. Both male hemizygous and female heterozygous A140V mice have lived to greater than 14 months of age with no unusual health issues.

### Increase in cell packing density in mutant animals

**Figure 5:**
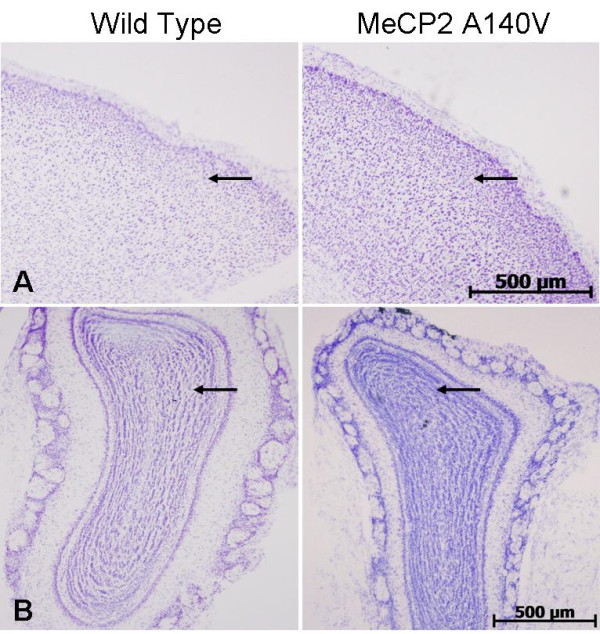
**Increases in cell density were found in the frontal cortex and olfactory bulbs of MeCP2 A140V mice**. Brain slices from WT and A140V male mice were stained with cresyl violet. Shown are areas of the frontal cortex (A) and olfactory bulbs (B). Arrows indicate areas of increased cell density. Scale bars are 500 μm.

**Figure 6:**
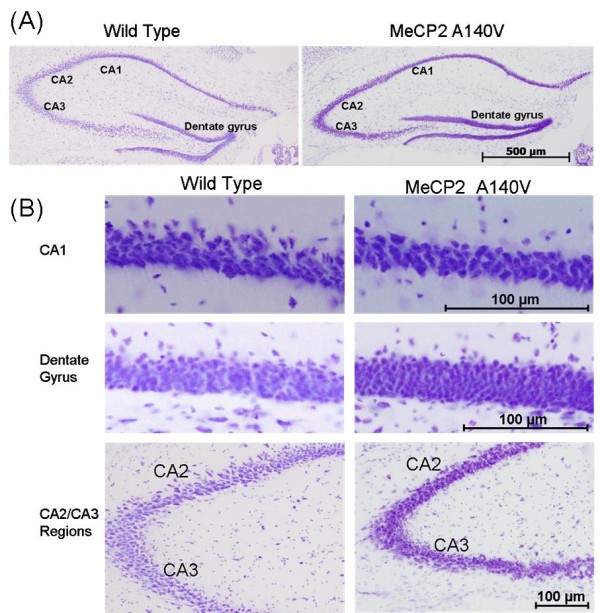
**Increases in cell density were found in the hippocampus and dentate gyrus of MeCP2 A140V mice**. Panel A shows cresyl violet stained hippocampi from WT and A140V mice. Staining is darker in the A140V mice due to an increase in cell density. Scale bar is 500 μm. Panel B contains enlargements of the CA1, dentate gyrus and CA2/CA3 regions of the hippocampi more clearly demonstrating the increase in cell density seen in Panel A. Scale bars in the enlarged images indicate 100 μm.

**Figure 7:**
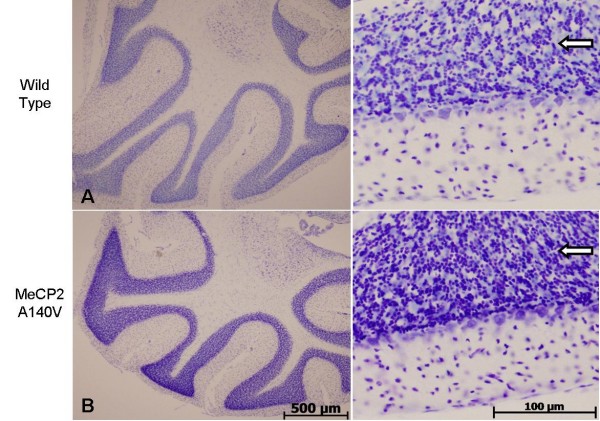
**Increases in cell density were found in the cerebellum of MeCP2 A140V mice**. Left panels show low power images of cresyl violet stained cerebelli from WT and A140V mice. The A140V cerebellum has noticeably darker staining due to an increase in cell density. Scale bar is 500 μm. Right panels show enlargement of these images demonstrating the differences in cell density. Scale bar is 100 μm. Arrows indicate areas of increased cell density.

**Figure 8:**
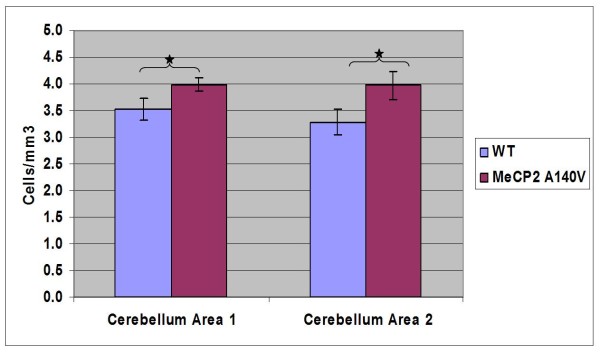
**Quantitative analysis of cell density in the cerebellum of MeCP2 A140V mice**. Cresyl violet stained cell nuclei in cerebellar sections from WT and A140V mice were counted in two separate areas of the granule cell layer in the cerebellum. In each cerebellar area, three adjacent regions were counted and the mean taken. The means for each area for each genotype are shown on the bar graph. The differences between the means were significant, (*) p < 0.05, indicating an increase in cell density in the A140V cerebellum.

We compared histological sections of brain tissue from 5 month old WT and A140V male mice (Cresyl violet staining). Although the WT and A140V sections were processed together, there was an apparent increase in staining intensity in the A140V mutant tissues. On closer examination, it became apparent that this was due to an increase in the packing density of cells in the mutant animals. This increase in packing density was noted in the frontal cortex and olfactory bulbs (Figure 5A and 5B), in the dentate gyrus and regions CA1, CA2, and CA3 of the hippocampus (Figure 6A and 6B), and in the granule cell layer of the cerebellum (Figure 7). The difference in cell packing density between A140V and WT male mice was quantified by counting all nuclei in a standard volume of tissue (80 mm^3^) from the cerebellar granule cell layer (Figure 8). This analysis demonstrated that there is a significantly higher (*p *< 0.05) density of cell nuclei in the A140V cerebellar granule layer than in WT controls.

### Abnormal dendritic branching in mutant cortical neurons

**Figure 9:**
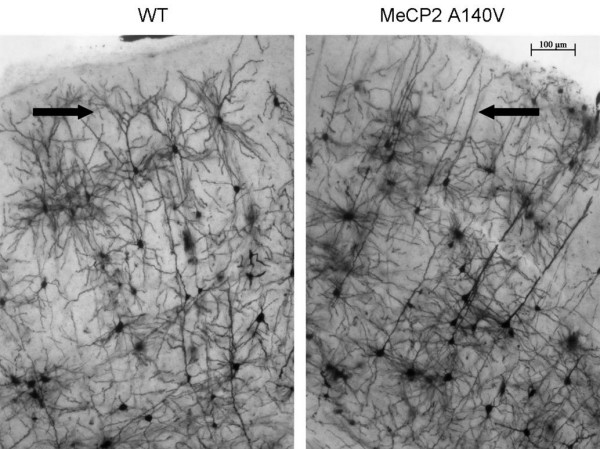
**Qualitative abnormalities of neuronal dendritic branching were seen in the layer II/III pyramidal neurons of the visual cortex of MeCP2 A140V mice**. Extended focus images of Golgi-Cox stained brain sections from WT and A140V mice are shown. Arrows indicate areas of apparent decreased dendritic branching in the A140V mouse in comparison to the WT mouse. Scale bar is 100 μm.

**Figure 10:**
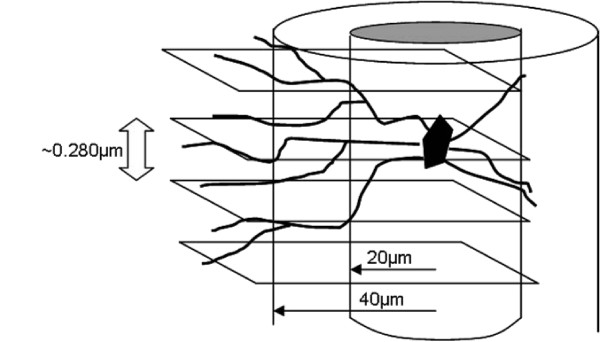
**The Scholl analysis technique for assessing dendritic complexity was adapted for use in brightfield Z-stack images of Golgi-Cox stained neurons**. The diagram shows an illustration of Scholl lines superimposed at 20 μm increments on a Z-stack of images of a stained neuron. This was done using Zeiss AxioVision software. Each Z-stack image is separated by only 0.280 μm allowing all dendritic intersections with Scholl lines to be clearly seen and counted. Drawing not to scale.

**Figure 11:**
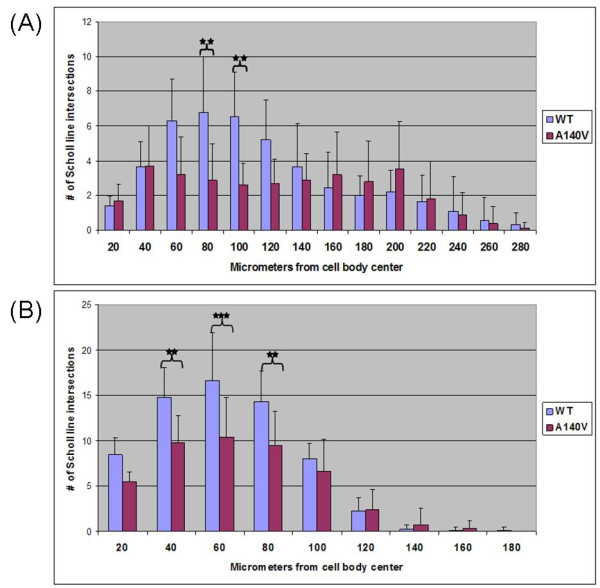
**Dendritic complexity of pryamidal neurons in the somatosensory cortex is decreased in MeCP2 A140V mice**. Scholl analysis was performed on Z-stack images of Golgi-Cox stained layer II-III pyramidal neurons from the somatosensory cortex of WT and A140V mice. Panel (A) shows the results of analysis of apical dendrites. Panel (B) shows the results of analysis of basal dendrites. Error bars indicate one standard deviation. (**) p < 0.01 (***) p < 0.001

Qualitative abnormalities of dendritic branching were noted upon initial visualization of various regions of Golgi-Cox stained cortical tissue from MeCP2 A140V mice. An extended focus (compressed Z-stack) image of the visual cortex is shown as an example (Figure 9). In order to begin to evaluate neuronal dendritic structure in the A140V mouse, Scholl analysis [46] was performed on layer II/III pyramidal neurons of the somatosensory cortex. This analysis was done using Scholl analysis on brightfield Z stack images of Golgi-Cox stained neurons in order to analyze the full arbors of both the apical and basal dendrites. A diagram of this adaptation of the Scholl analysis technique is shown in Figure 10. Male A140V and wild type mice 2-3 months old were used for this analysis. This quantitative analysis demonstrated significant differences in the branching complexities of the dendritic arbors of these neurons. Apical dendrites showed very significant differences in branching at 80 and 100 μm distances from the cell body and basal dendrites showed significant differences at 40, 60, and 80 μm (Figure 11A and 11B).

## Discussion

The A140V mouse model was constructed in order to more closely examine the mental retardation associated with RTT. This was expected to be a mental retardation model in mice due to the effects of this mutation in human male patients. Male patients with the A140V mutation survive into adulthood, indicating that this mutation is less likely to compromise functions essential for life. However, significant mental retardation is common in males carrying this mutation while carrier females show mild or no symptoms [23-26]. The A140V mutation is predicted to be non-truncating and this was verified by immunoflourescence staining of brain slices from WT and MeCP2 A140V male mice. The staining was performed with a C-terminal MeCP2 antibody to demonstrate that the MeCP2 protein was not truncated by the A140V mutation.

The initial characterization of the MeCP2 A140V mouse has revealed some interesting abnormalities. During casual observation the mice appear normal with no variation in weight or body morphology and no indications of seizures or tremors. However, there are cellular abnormalities in the brain consistent with those seen in RTT syndrome and other diseases resulting in mental retardation. An increase in cell packing density was apparent upon histological studies of brain tissue from the mutant animals. Qualitative differences in cell density in the cerebellum were confirmed by performing cell counts. The results of these cell counts showed statistically significant differences in cell density. Increased cell packing density has been previously noted in both human RTT patients [5] and in RTT mouse models [39,44,45]. The MeCP2 null mouse created by deletion of exon 3 revealed increases in cell density in the hippocampus, cerebral cortex, and cerebellum that closely parallel our own results [39]. A study of the brains of three RTT patients found an increase in cell-packing density in multiple brain regions including the cerebral cortex, basal ganglia, thalamus, hippocampus, and amygdala. Abnormalities were also reported in the cerebellum although an increase in cell packing density was not reported [5].

We find no previous descriptions of cell density abnormalities in the olfactory bulbs of Rett mouse models. We are uncertain if this is because cell density abnormalities have not previously been seen in olfactory bulbs or if the olfactory bulbs were not examined in these mice. There have been reports of defects in neuronal development in olfactory neurons from the nasal epithelium of RTT patients [47]. In addition, defects of olfactory neuron maturation and protein expression have been found in two previously described RTT mouse models [48-51], indicating that olfactory defects are not unusual in RTT or in RTT mouse models. In addition, a recent and very thorough study of brain morphology found changes in the volume and shape of olfactory bulbs in two MeCP2 null mouse models [43]. We have seen abnormalities in the shape of olfactory bulbs and the morphology of glomeruli in some the A140V male hemizygous mice although this has not been quantified and will be the subject of further study.

A lack of branching complexity in cortical pyramidal neurons in many brain regions has been reported in human cases of RTT [6-9] as well as in mouse models [44,45]. In fact, dendritic branching anomalies are often found in conjunction with mental retardation of many causes [52,53]. The A140V mutation is strongly associated with mental retardation [23-26]; therefore, we felt that abnormal dendritic branching patterns of neurons would likely occur in this new mouse model. We explored the branching complexity of neurons in the A140V mouse model and found differences between WT and MeCP2 A140V mice. We noted significant differences in the branching complexity of both the apical and basal dendrites of layer II/III pyramidal neurons of the somatosensory cortex as measured by Scholl analysis. This correlates closely with other reports of decreases in branching complexity of cortical pyramidal neurons in tissue from both human RTT patients and in *Mecp2 *mutant mouse models. We also found apparent differences in branching complexity in the visual cortex although this has not yet been quantified.

The MeCP2 A140V mouse has proven to be distinct from other mouse models of MeCP2 mutation. Like its human counterpart, the A140V mutation in mice produces symptoms both correlating with and disparate from the classical symptoms of RTT. In humans, the A140V mutation causes a mental retardation syndrome that has associated with it abnormalities of gait, kyphoscoliosis, mild microcephaly, spasticity, dysarthric speech, and occasionally mental illness such as schizophrenia [21,23,26,27]. However certain features of classic Rett syndrome such as stereotypic hand movements, breathing abnormalities and seizures [2,3] are notably absent in humans with the A140V mutation. These distinctions suggest that the A140V mutation affects certain functions of the MeCP2 protein while preserving others. There is for instance the report indicating that the MeCP2 A140V protein has an increased binding affinity for unmethylated DNA [30], the functional consequences of which are unknown. There is also the interesting finding that although the A140V mutation preserves the methyl-CpG binding function of MeCP2 it does interfere with binding to the mental retardation associated protein ATRX [29]. This raises the possibility that disruption of the MeCP2-ATRX interaction is in someway related to the neurological symptoms that occur in both humans with the A140V mutation and in our engineered mice.

ATRX is chromatin remodeling protein that is a SWI2/SNF2 DNA helicase ATPase that is the cause of ATRX syndrome [33]. This protein has only recently been identified as a binding partner of the MeCP2 protein. Analysis of brain tissue from MeCP2-null mice revealed that loss of MeCP2 in mature neurons leads to delocalization of the ATRX protein, suggesting that MeCP2 may be required for the proper localization of ATRX. *In vitro *studies done using fusion proteins in mouse fibroblasts revealed that the MeCP2 A140V protein, while properly localized to heterochromatin within the nucleus, fails to recruit ATRX to the heterochromatin. In contrast, WT MeCP2 protein efficiently recruits ATRX to heterochromatin [29]. This failure to localize ATRX to heterochromatin may be important in understanding the normal function of both the MeCP2 and ATRX proteins.

We believe that it is important to note that the mice used in these experiments belong to early generations (F1 to F4) and are therefore in a mixed 129X1/S1 and C57BL/6 background. In addition, the neomycin resistance gene cassette used in the construction of these mice has not been excised from the experimental animals although we have begun production of a line of mice in which the neo cassette has been removed. As genetic background and the presence of extra DNA sequences such as the neo cassette can contribute to phenotype we will be repeating these experiments in later generation mice lacking the neo cassette in order to confirm our experimental results. However, we believe that, given the close parallels of our data with that seen in other mouse models of *Mecp2 *gene disruption, the phenotype we have observed is in fact due to the A140V mutation itself.

## Conclusions

We believe that this new mouse model will provide insight into the functions of MeCP2, including its association with the ATRX protein. The A140V mouse has proven to reproduce some of the abnormalities of other *Mecp2 *mouse models (increased cell packing density and decreased neuronal dendritic arbors) while failing to reproduce others (seizures, tremors, kyphosis, breathing abnormalities, shortened life span). With this knowledge we can continue dissecting the functions of MeCP2 in the hopes of explaining its relationship to mental retardation and other clinical symptoms.

## Methods

### Generation of A140V "knock-in" mice

Breeding protocols and all animal experiments were approved by the Institutional Animal Care and Use Committee (IACUC) of St. Joseph's Hospital and Medical Center. An 11 kbp Bam HI fragment of the mouse MeCP2 gene, that included exon 3 and part of exon 4, was isolated by restriction digestion of a BAC clone (mBAC B22804). The 5'- end of the insert was first trimmed by restriction digestion with Pml I. A pGK-Neo cassette (flanked by two Frt sites and a single LoxP site, gift of Dr. Dymecki (Department of Genetics, Harvard Medical School), was inserted into the Nde I site located about 1.7 kbp upstream of exon 3. A second LoxP site and a novel Acl I restriction were inserted into the 3'-UTR of exon 4 (into an Mfe I site located about 400 bp downstream of the translation termination codon). A single base mutation was introduced using the QuikChangeXL kit (Stratagene Inc., Cedar Creek, Texas): a C to T change at nucleotide position 601 of the *Mecp2-e2 *mRNA, resulting in the A140V missense mutation. Pml I linearized targeting vector was electroporated into the R1 line of ES cells [54] (male genotype, Strain 129X1/S1) under previously described conditions [55]. G418 (neomycin analogue) resistant clones were tested for proper recombination at the 3'-integration site by a PCR based assay using the following primers:

F30: 5' CGACAAGCACAGTCAGGTTGAAGAC 3'

B34: 5' TCCCAAGAGTCCCATAGTTTCTCC 3'

The wild-type allele yields an amplimer that is 322 bp long, while the mutant allele yields an amplimer that is 362 bp long. Random integration of the targeting vector into the mouse genome results in both wild-type and mutant bands. The PCR product was also digested with Acl I, which cuts the mutant amplimer into two segments (227 bp and 135 bp), leaving the wild-type amplimer uncut. Selected ES clone DNAs were tested by Southern blot for correct recombination at the 5'end, using Probe A. Homologous recombination at the 5' end results in a 5.5 kbp band, and clones that retain the wild-type locus produce an 11 kbp band. Finally, selected positive ES cell clones were verified by sequencing to confirm the presence of the desired point mutation. Two ES cell clones (169J6 and 177U9) were then injected into blastocysts from C57BL/6 mice (done under contract by Ingenious Targeting Laboratories, Inc., Stony Brook, New York) and implanted into pseudo-pregnant females. Male chimeras were harem bred with wild-type C57BL/6 mice and tail DNAs from all female F1 pups were tested for the presence of the transgene. Founder females (derived from ES clone 177U9) were identified by PCR, Southern blot, and DNA sequencing. These founder females were used to establish our colony of A140V mice by backcrossing with WT C57BL/6NCrL male mice (Charles River, Wilmington, Massachusetts), yielding mutant hemizygous males, heterozygous females, and WT controls. Heterozygous females produced in the colony were backcrossed with WT C57BL/6NCrL male mice to produce experimental mice as well as additional breeding stock.

### MeCP2 RT-PCR

Total RNA was extracted from the cerebella of 2 mutant and 2 WT male mice and treated with DNase I to remove traces of genomic DNA. cDNA was synthesized using random hexamers (Superscript III kit, Invitrogen, Inc., Carlsbad, California). cDNA from each of these four animals was used in PCR reactions with the F30 and B34 primers described above. The amplimer from mutant animals is 40 bp larger than the amplimer from WT animals. A second cDNA fragment (729 bp) that included a portion of exon 3 and exon 4, containing the A140V point mutation was then amplified using the following primers:

U256 (located within exon 3): 5' GTATGATGACCCCACCTTGC 3'

L1452 (located within exon 4): 5' TTCAGTCCCTTCCCGCTTTT 3'

The cDNA amplimers from WT and mutant RNAs were gel purified and sequenced to verify WT or mutant status at the codon for amino acid 140. When genomic DNA was used as a template for PCR using these same primers, the amplimer was 1216 bp long (providing a control for contamination of the RNA by genomic DNA).

### qRT-PCR

Total RNA was extracted from cerebella of three mutant male mice and three WT male littermates (6 months old) using Trizol (Invitrogen, Inc., Carlsbad, California) and cleaned up using an RNA extraction kit (SA Biosciences, Frederick, MD). First strand cDNA was synthesized (kit from SA Biosciences) and used in qRT-PCR reactions using PCR assays and SYBR Green master mix from SA Biosciences (*Mecp2*, cat no. PPM31215E, and *Gapdh*, cat no. PPM02946E). Controls were first done with mouse reference RNA to verify comparable efficiency of PCR reactions, and negative controls included no-reverse transcriptase (NRT) reactions for each RNA. Triplicate reactions were done for each primer assay and each of the 6 cDNA samples. Threshold level of fluorescence was set across the entire run within the exponential phase of amplification. The corresponding C_t _values were then determined for each sample in the run. The means, standard deviations, and *p *value were calculated using GraphPad InStat3 software.

### Histology: Cresyl Violet Staining of Brain Tissue

Mice were anesthetized and transcardially perfused with saline followed by 4% buffered paraformaldehyde (PFA). The brains were then post-fixed in 4% PFA overnight at 4°C and then cryoprotected in 30% sucrose for 1-2 days. Subsequently, brains were frozen in an isopentane/dry ice bath. The brains were immediately sectioned into 30 μm slices using a cryostat, slices were placed on slides, and then kept frozen at -20°C until staining. Alternating brain slices were then thawed briefly and stained with cresyl violet (Sigma, St. Louis, Missouri), cover slipped with Permount (Fisher Scientific, Pittsburgh, PA) and were visualized with either an Olympus microscope or a Zeiss AxioImager microscope (for brightfield Z-stack images).

### Cell Nuclei Count Analysis

Cell nuclei counts were performed in the cerebellum (using brightfield z-stack microscopy) in order to quantify differences in cell nuclei number. This was done using brightfield Z-stack images of the granule cell layer of the cerebellum in cresyl violet stained tissue. A rectangular counting area was drawn on the Z-stack images using Axiovision software. This demarcated area carried through the entire Z-stack. Calculating the distance between the top and bottom slices of the stack in conjunction with the demarcated area allowed for a volume measurement to be made. All cell nuclei counts were done in a total volume of 80 mm^3^. Using this counting method, nuclei were clear and easily distinguishable from each other. Triplicate cell counts were performed in each region of the cerebellar granule cell layer from WT and MeCP2 A140V mice. The mean, standard deviation and *p *values were calculated using GraphPad InStat3 software.

### Histology: Golgi-Cox Staining of Brain Tissue

The Rapid Golgi-Cox staining kit (FD Neurotechnologies, Ellicott City, Maryland) was used for staining whole mouse brains. Mice were euthanized by overdose of isofluorane and the brains removed. After rinsing in de-ionized water the brains were incubated for 2 weeks in a Solution A/B mixture (impregnation) following the manufacturer's protocol. The brains were then moved to Solution C (cryoprotection) for 4-5 days and subsequently frozen in an isopentane/dry ice bath. The brains were immediately sectioned into 100-200 μm slices using a cryostat and the slices were mounted on gelatin-coated slides. After being allowed to dry for 3-5 days, the staining protocol for development of the black precipitate was completed using kit reagents. The tissue was counterstained with cresyl violet (Sigma, St. Louis, Missouri). Cover slips were mounted using Permount (Fisher Scientific, Pittsburgh, Pennsylvania) and slides were visualized using a Zeiss AxioImager microscope capable of brightfield Z-stack.

### Scholl Analysis

Scholl analysis [46] was adapted for use on brightfield Z-stack images using Axiovision software. Concentric circles (Scholl lines) were drawn at 20 μm increments from the center of the cell body. The Scholl lines were superimposed on the entire stack of images allowing the analysis to be conducted in a 3-dimensional fashion. The dendritic arbor was followed through the Z-stack and the number of intersections made by dendrites with the Scholl lines was measured as each dendrite traveled away from the cell body. The Z-stack image slices were separated by a depth of only 0.280 μm preventing intersections from being missed.

The apical and basal arbors of 10 neurons from a MeCP2 A140V mouse and 10 neurons from a WT mouse were captured in Z-stack images. All neurons were layer II/III pyramidal neurons of similar morphology, located in the somatosensory cortex at similar coordinates [56]. Scholl lines were placed on the images every 20 μm until no more dendrites crossed the Scholl lines. Axiovision software was then used to manually mark crossings of each Scholl line by the dendrites of the neurons. Crossings at each Scholl line were summed and the mean and standard deviation calculated. *p *values comparing the wild type and A140V mouse neurons at each Scholl line were then determined. GraphPad InStat3 software was used for statistical analysis.

### Immunohistochemistry

Brain tissue from WT and A140V mutant mice was frozen in an isopentane/dry ice bath and 10 μm frozen sections were cut on a cryostat, placed on slides, and frozen at -20°C until staining. Tissue was then fixed with 4% PFA, blocked and permeabilized with a mixture of 10% goat serum, 1% BSA, and 0.2% Triton X100, and incubated overnight at 4°C with anti-MeCP2 antibody (PA1-888 rabbit anti-MeCP2 antibody, Affinity BioReagents, Rockford, Illinois). This is a polyclonal antibody that recognizes the C-terminus of the MeCP2 protein. Sections were then incubated with secondary antibody (Alexa Fluor 488 goat anti-rabbit antibody, Invitrogen, Carlsbad, California) for 2 hours at room temperature. Following a wash step, sections were coverslipped with Pro-long Gold (Invitrogen, Carlsbad, California) mounting medium. MeCP2 staining in the slices was visualized using a Zeiss confocal microscope.

## Authors' contributions

VN, GH and SO constructed and verified the mouse model. SO genotyped and maintained the mouse colony. GJ performed the experiments. SR assisted with tissue processing and microscope technique. NS provided fresh frozen mouse brain slices and tested antibodies for immunohistochemistry. GJ and VN wrote the manuscript with editing from GH, SN, and SR. MB provided experimental advice as well as MeCP2 null mouse tissue for experimental controls. All authors read and reviewed the final manuscript.

## References

[B1] Amir RE Veyver IB Van Den Wan M Tran CQ Franke U Zoghbi HY Rett syndrome is caused by mutations in X-linked *MECP2*, encoding methyl-CpG-binding protein 2 Nature Genetics 1999 23 185 188 10.1038/13810 10508514

[B2] Hagberg B Aicardi J Dias K Ramos O A progressive syndrome of autism, dementia, ataxia, and loss of purposeful hand use in girls: Rett's syndrome: report of 35 cases Annals of Neurology 1983 14 471 479 10.1002/ana.410140412 6638958

[B3] Hagberg B Rett's Syndrome: Prevalence and Impact on Progressive Severe Mental Retardation in Girls Acta Paediatrica Scandinavica 1985 74 405 408 10.1111/j.1651-2227.1985.tb10993.x 4003065

[B4] Dunn HG MacLeod PM Rett syndrome: review of biological abnormalities Can J Neurol Sci 2001 28 16 29 11252289 10.1017/s0317167100052513

[B5] Bauman ML Kemper TL Arin DM Pervasive neuroanatomic abnormalities of the brain in three cases of Rett's syndrome Neurology 1995 45 1581 1586 7644058 10.1212/wnl.45.8.1581

[B6] Belichenko PV Rett syndrome: 3-D confocal microscopy of cortical pyramidal dendrites and afferents NeuroReport 1994 5 1509 1513 10.1097/00001756-199407000-00025 7948850

[B7] Armstrong D Dunn JK Antalffy B Trivedi R Selective dendritic alterations in the cortex of Rett syndrome Journal of Neuropathology and Experimental Neurology 1995 54 195 201 10.1097/00005072-199503000-00006 7876888

[B8] Armstrong DD Neuropathology of Rett syndrome J Child Neurol 2005 20 747 753 10.1177/08830738050200082401 16225830

[B9] Armstrong DD Dunn K Antalffy B Decreased dendritic branching in frontal, motor and limbic cortex in Rett syndrome compared with trisomy 21 J Neuropathol Exp Neurol 1998 57 1013 1017 10.1097/00005072-199811000-00003 9825937

[B10] Neul JL Zoghbi HY Rett syndrome: a prototypical neurodevelopmental disorder Neuroscientist 2004 10 118 128 10.1177/1073858403260995 15070486

[B11] Nan X Campoy FJ Bird A MeCP2 is a transcriptional repressor with abundant binding sites in genomic chromatin Cell 1997 88 471 481 10.1016/S0092-8674(00)81887-5 9038338

[B12] Nan X Tate P Li E Bird A DNA methylation specifies chromosomal localization of MeCP2 Mol Cell Biol 1996 16 414 421 8524323 10.1128/mcb.16.1.414 PMC231017

[B13] Chahrour M Jung SY Shaw C Zhou X Wong ST Qin J Zoghbi HY MeCP2, a key contributor to neurological disease, activates and represses transcription Science 2008 320 1224 1229 10.1126/science.1153252 18511691 PMC2443785

[B14] Jones PL Veenstra GJ Wade PA Vermaak D Kass SU Landsberger N Strouboulis J Wolffe AP Methylated DNA and MeCP2 recruit histone deacetylase to repress transcription Nat Genet 1998 19 187 191 10.1038/561 9620779

[B15] Sun YE Wu H The ups and downs of BDNF in Rett syndrome Neuron 2006 49 321 323 10.1016/j.neuron.2006.01.014 16446133

[B16] Zhou Z Hong EJ Cohen S Zhao W Ho HH Schmidt L Chen WG Lin Y Savner E Griffith EC Brain-specific phosphorylation of MeCP2 regulates activity-dependent *Bdnf *transcription, dendritic growth, and spine maturation Neuron 2006 52 255 269 10.1016/j.neuron.2006.09.037 17046689 PMC3962021

[B17] Poo MM Neurotrophins as synaptic modulators Nat Rev Neurosci 2001 2 24 32 10.1038/35049004 11253356

[B18] Chang Q Khare G Dani V Nelson S Jaenisch R The disease progression of *Mecp2 *mutant mice is affected by the level of BDNF expression Neuron 2006 49 341 348 10.1016/j.neuron.2005.12.027 16446138

[B19] Meloni I Bruttini M Longo I Mari F Rizzolio F D'Adamo P Denvriendt K Fryns JP Toniolo D Renieri A A mutation in the rett syndrome gene, MECP2, causes X-linked mental retardation and progressive spasticity in males Am J Hum Genet 2000 67 982 985 10.1086/303078 10986043 PMC1287900

[B20] Imessaoudene B Bonnefont JP Royer G Cormier-Daire V Lyonnet S Lyon G Munnich A Amiel J MECP2 mutation in non-fatal, non-progressive encephalopathy in a male J Med Genet 2001 38 171 174 10.1136/jmg.38.3.171 11238684 PMC1734835

[B21] Couvert P Bienvenu T Aquaviva C Poirier K Moraine C Gendrot C Verloes A Andres C Le Fevre AC Souville I MECP2 is highly mutated in X-linked mental retardation Hum Mol Genet 2001 10 941 946 10.1093/hmg/10.9.941 11309367

[B22] Bienvenu T Chelly J Molecular genetics of Rett syndrome: when DNA methylation goes unrecognized Nature Reviews: Genetics 2006 7 415 426 10.1038/nrg1878 16708070

[B23] Dotti MT Orrico A De Stephano N Battisti C Sicurelli F Severi S Lam CW Galli L Sorrentino V Federico A A Rett syndrome MECP2 mutation that causes mental retardation in men Neurology 2002 58 226 230 11805248 10.1212/wnl.58.2.226

[B24] Klauck SM Lindsay S Beyer KS Splitt M Burn J Poustka A A mutation hot spot for nonspecific X-linked mental retardation in the *MECP2 *gene causes the PPM-X syndrome American Journal of Medical Genetics 2002 70 1034 1037 10.1086/339553 PMC379098 11885030

[B25] Orrico A Lam C Galli L Dotti MT Hayek G Tong SF Poon PM Zappella M Federico A Sorrentino V MECP2 mutation in male patients with non-specific X-linked mental retardation FEBS Lett 2000 481 285 288 10.1016/S0014-5793(00)01994-3 11007980

[B26] Winnepenninckx B Errijgers V Hayez-Delatte F Reyniers E Kooy RF Identification of a family with nonspecific mental retardation (MRX79) with the A140V mutation in the *MECP2 *gene: Is there a need for routine screening? Human Mutation 2002 20 249 252 10.1002/humu.10130 12325019

[B27] Cohen D Lazar G Couvert P Desportes V Lippe D Mazet P Heron D MECP2 mutation in a boy with language disorder and schizophrenia Am J Psychiatry 2002 159 148 149 10.1176/appi.ajp.159.1.148-a 11772708

[B28] Ohki I Shimotake N Fujita N Jee J Ikegami T Nakao M Shirakawa M Solution structure of the methyl-CpG binding domain of human MBD1 in complex with methylated DNA Cell 2001 105 487 497 10.1016/S0092-8674(01)00324-5 11371345

[B29] Nan X Hou J Maclean A Nasir J Lafuente MJ Shu X Kriaucionis S Bird A Interaction between chromatin proteins MECP2 and ATRX is disrupted by mutations that cause inherited mental retardation Proceedings of the National Academy of Sciences USA 2007 104 2709 2714 10.1073/pnas.0608056104 PMC1796997 17296936

[B30] Kudo S Nomura Y Segawa M Fujita N Nakao M Hammer S Schanen C Terai I Tamura M Functional characterisation of MeCP2 mutations found in male patients with X linked mental retardation J Med Genet 2002 39 132 136 10.1136/jmg.39.2.132 11836365 PMC1735040

[B31] Stevenson RE Alpha-Thalassemia X-Linked Mental Retardation Syndrome GeneReviews 2009 http://www.ncbi.nlm.nih.gov/bookshelf/br.fcgi?book=gene&part=xlmr

[B32] Villard L Fontes M Alpha-thalassemia/mental retardation syndrome, X-linked (ATR-X, MIM #301040, ATR-X/XNP/XH2 gene MIM #300032 European Journal of Human Genetics 2002 10 223 225 10.1038/sj.ejhg.5200800 12032728

[B33] Gibbons RJ Higgs DR Molecular-clinical spectrum of the ATR-X syndrome American Journal of Medical Genetics 2000 97 204 212 10.1002/1096-8628(200023)97:3<204::AID-AJMG1038>3.0.CO;2-X 11449489

[B34] Badens C Lacoste C Philip N Martini N Courrier S Giuliano F Verloes A Munnich A Leheup B Burglen L Mutations in PHD-like domain of the *ATRX *gene correlate with severe psychomotor impairment and severe urogenital abnormalities in patients with ATRX syndrome Clinical Genetics 2006 70 57 62 10.1111/j.1399-0004.2006.00641.x 16813605

[B35] Garrick D Sharpe JA Arkell R Dobbie L Smith AJ Wood WG Higgs DR Gibbons RJ Loss of Atrx affects trophoblast development and the pattern of X-inactivation in extraembryonic tissues PLoS Genet 2006 2 e58 10.1371/journal.pgen.0020058 16628246 PMC1440874

[B36] Berube NG Mangelsdorf M Jagla M Vanderluit J Garrick D Gibbons RJ Higgs DR Slack RS Picketts DJ The chromatin-remodeling protein ATRX is critical for neuronal survival during corticogenesis J Clin Invest 2005 115 258 267 15668733 10.1172/JCI22329 PMC544602

[B37] Medina CF Mazerolle C Wang Y Berube NG Coupland S Gibbons RJ Wallace VA Picketts DJ Altered visual function and interneuron survival in Atrx knockout mice: inference for the human syndrome Hum Mol Genet 2009 18 966 977 10.1093/hmg/ddp034 19088125

[B38] Guy J Hendrich B Holmes M Martin JE Bird A A mouse *Mecp2 *-null mutation causes neurolgical symptoms that mimic Rett syndrome Nature Genetics 2001 27 322 326 10.1038/85899 11242117

[B39] Chen RZ Akbarian S Tudor M Jaenisch R Deficiency of methyl-CpG binding protein-2 in CNS neurons results in a Rett-like phenotype in mice Nature Genetics 2001 27 327 331 10.1038/85906 11242118

[B40] Shahbazian MD Young JI Yuva-Paylor LA Spencer CM Antalffy BA Noebels JL Armstrong DL Paylor R Zoghbi HY Mice with truncated MeCP2 recapitulate many Rett syndrome features and display hyperacetylation of histone H3 Neuron 2002 35 243 254 10.1016/S0896-6273(02)00768-7 12160743

[B41] McGill BE Bundle SF Yaylaoglu MB Carson JP Thaller C Zoghbi HY Enhanced anxiety and stress-induced corticosterone release are associated with increased Crh expression in a mouse model of Rett syndrome Proc Natl Acad Sci USA 2006 103 18267 18272 10.1073/pnas.0608702103 17108082 PMC1636379

[B42] Lawson-Yuen A Liu D Han L Jiang ZI Tsai GE Basu AC Picker J Feng J Coyle JT Ube3a mRNA and protein expression are not decreased in Mecp2^R168X ^mutant mice Brain Research 2007 1180 1 6 10.1016/j.brainres.2007.08.039 17936729 PMC2706140

[B43] Belichenko NP Belichenko PV Li HH Mobley WC Francke U Comparative study of brain morphology in Mecp2 mutant mouse models of Rett syndrome J Comp Neurol 2008 508 184 195 10.1002/cne.21673 18306326

[B44] Fukuda T Itoh M Ichikawa T Washiyama K Goto Y Delayed maturation of neuronal architecture and synaptogenesis in cerebral cortex of Mecp2-deficient mice J Neuropathol Exp Neurol 2005 64 537 544 15977646 10.1093/jnen/64.6.537

[B45] Kishi N Macklis JD MECP2 is progressively expressed in post-migratory neurons and is involved in neuronal maturation rather than cell fate decisions Mol Cell Neurosci 2004 27 306 321 15519245 10.1016/j.mcn.2004.07.006

[B46] Scholl DA Dendritic organization in the neurons of the visual and motor cortices of the cat Journal of Anatomy 1953 87 387 406 13117757 PMC1244622

[B47] Ronnett GV Leopold D Cai X Hoffbuhr KC Moses L Hoffman EP Naidu S Olfactory biopsies demonstrate a defect in neuronal development in Rett's syndrome Ann Neurol 2003 54 206 218 10.1002/ana.10633 12891673

[B48] Degano AL Pasterkamp RJ Ronnett GV MeCP2 deficiency disrupts axonal guidance, fasciculation, and targeting by altering Semaphorin 3F function Mol Cell Neurosci 2009 42 243 254 10.1016/j.mcn.2009.07.009 19628041 PMC3290450

[B49] Matarazzo V Cohen D Palmer AM Simpson PJ Khokhar B Pan SJ Ronnett GV The transcriptional repressor Mecp2 regulates terminal neuronal differentiation Mol Cell Neurosci 2004 27 44 58 10.1016/j.mcn.2004.05.005 15345242

[B50] Matarazzo V Ronnett GV Temporal and regional differences in the olfactory proteome as a consequence of MeCP2 deficiency Proc Natl Acad Sci USA 2004 101 7763 7768 10.1073/pnas.0307083101 15128950 PMC419680

[B51] Palmer A Qayumi J Ronnett G *MeCP2 *mutation causes distinguishable phases of acute and chronic defects in synaptogenesis and maintenance, respectively Molecular and Cellular Neuroscience 2008 37 794 807 10.1016/j.mcn.2008.01.005 18295506

[B52] Machado-Salas JP Abnormal dendritic patterns and aberrant spine development in Bourneville's disease--a Golgi survey Clin Neuropathol 1984 3 52 58 6713754

[B53] Kaufmann WE Moser HW Dendritic anomalies in disorders associated with mental retardation Cerebral Cortex 2000 10 981 991 10.1093/cercor/10.10.981 11007549

[B54] Nagy A Rossant J Nagy R Abramow-Newerly W Roder JC Derivation of completely cell culture-derived mice from early-passage embryonic stem cells Proc Natl Acad Sci USA 1993 90 8424 8428 10.1073/pnas.90.18.8424 8378314 PMC47369

[B55] Homanics GE Ferguson C Quinlan JJ Daggett J Snyder K Lagenaur C Mi ZP Wang XH Grayson DR Firestone LL Gene knockout of the alpha6 subunit of the gamma-aminobutyric acid type A receptor: lack of effect on responses to ethanol, pentobarbital, and general anesthetics Mol Pharmacol 1997 51 588 596 9106623 10.1124/mol.51.4.588

[B56] Paxinos G Franklin K The Mouse Brain in Stereotaxic Coordinates 2001 2 San Diego, CA 92101 USA: Academic Press

